# Blastic plasmacytoid dendritic cell neoplasm following Langerhans cell histiocytosis

**DOI:** 10.1016/j.jdcr.2026.04.042

**Published:** 2026-04-29

**Authors:** Marina Goto, Taro Akatsuka, Eri Uchiyama, Hiroi Urabe, Asuka Shibamiya, Chiaki Nakaseko, Mina Komuta, Yuichiro Hayashi, Toshihisa Hamada, Makoto Sugaya

**Affiliations:** a School of Medicine, International University of Health and Welfare, Narita, Chiba, Japan; b Department of Dermatology, International University of Health and Welfare, Narita, Chiba, Japan; c Department of Hematology, International University of Health and Welfare, Narita, Chiba, Japan; d Department of Diagnostic Pathology, International University of Health and Welfare, Narita, Chiba, Japan

**Keywords:** blastic plasmacytoid dendritic cell neoplasm, BRAF-V600E mutation, c-Myc, Langerhans cell histiocytosis, multipotent progenitors

## Introduction

Langerhans cell histiocytosis (LCH) is a hematopoietic neoplasm characterized by granulomatous lesions composed of clonal langerin-positive dendritic cells. It can affect various organs, including the skin, bone, lungs, and hematopoietic system. It is a rare disease, with an annual incidence of less than 1 case per 10 million adults.^1^ In adults, most cases with skin lesions have systemic involvement. It is extremely rare for patients to have only skin lesions.^2^

Blastic plasmacytoid dendritic cell neoplasm (BPDCN) is an aggressive myeloid neoplasm derived from precursors of plasmacytoid dendritic cells.^3^ The median overall survival is only several months. It typically presents with solitary or multiple skin lesions, followed by rapid systemic dissemination with leukemic involvement in most cases. BPDCN accounts for 0.44% of all hematologic malignancies^4^ and has an incidence of 4 cases per 10 million people.^5^

Both LCH and BPDCN are rare dendritic cell neoplasms derived from different dendritic cell lineages. The sequential occurrence of these 2 entities in the same patient is extremely rare. Such a case may provide insight into their pathogenesis and possible shared cellular origin. In addition, recognition of this association may have implications for long-term monitoring of patients with LCH. Here, we report a case of LCH followed by BPDCN. Immunohistochemical analysis suggested that the tumor cells in each disease had different molecular alterations despite a possible common precursor.

## Case report

**Fig 1:**
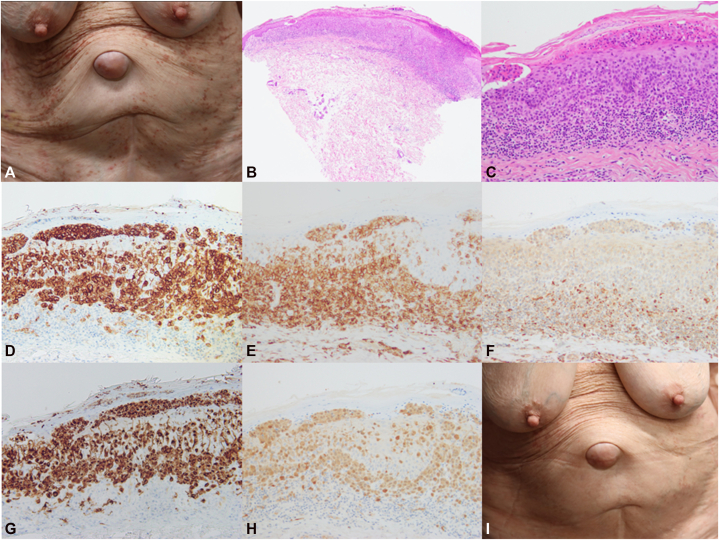
**A,** Multiple bean-sized scaly brown slightly indurated plaques on the trunk at the first visit. **B** and **C,** H&E staining of skin lesion (40× and 200×, respectively). Band-like infiltrate of mononuclear cells in the upper dermis. Infiltration and focal collections of mononuclear cells are also seen in the epidermis. Higher magnification showed infiltration of round cells with abundant eosinophilic cytoplasm and prominent nuclear grooves. Lymphocytic infiltrate is also seen. **D-H,** Immunohistological staining for CD1a **(D)**, CD4 **(E)**, CD68 **(F)**, langerin **(G)**, and S100 **(H)**. All pictures are at 200× magnification. **I,** Clearance of skin lesions 6 months after treatment with topical steroids and narrowband UVB phototherapy. *H&E*, hematoxylin and eosin; *UVB*, ultraviolet B.

A 78-year-old woman with polyclonal gammaglobulinemia presented with multiple, bean-sized, scaly, brown, slightly indurated plaques on the trunk that had developed 15 months earlier (Fig 1, *A*). Her medical history included chronic heart failure, atrial fibrillation, myocardial infarction, cardiac sarcoidosis, peripheral arterial occlusion of the lower extremities, and colon polyps. Treatment with topical steroids was ineffective. A skin biopsy revealed a band-like infiltrate of mononuclear cells in the upper dermis (Fig 1, *B*). Infiltration and focal collections of mononuclear cells were also seen in the epidermis. At higher magnification, round cells with abundant eosinophilic cytoplasm and prominent nuclear grooves were observed (Fig 1, *C*). A lymphocytic infiltrate was also present. Immunohistochemical staining showed that these cells were positive for CD1a, CD4, CD68, langerin, and S100 (Fig 1, *D-H*). Based on these findings, a diagnosis of LCH was made. Computed tomography and bone scintigraphy showed no involvement of other organs. Most skin lesions resolved 6 months after treatment with topical steroids and narrowband ultraviolet B phototherapy (Fig 1, *I*).

**Fig 2:**
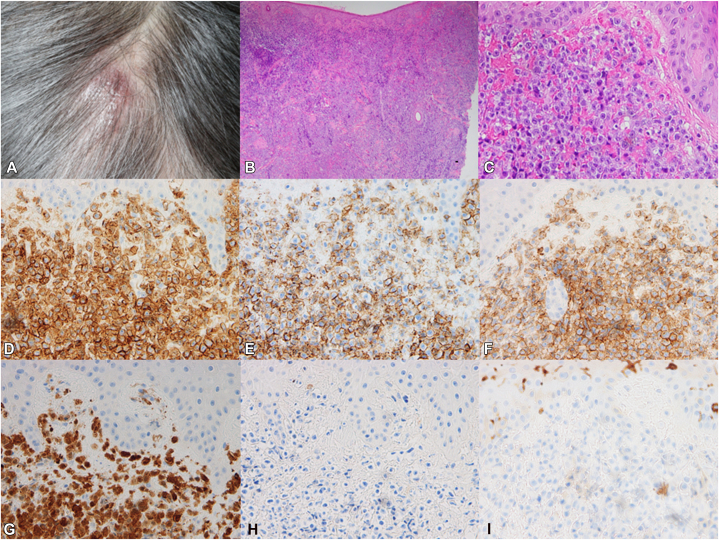
**A,** A reddish nodule on the scalp. **B** and **C,** H&E staining of skin lesion (40× and 400×, respectively). Dense infiltrate of lymphoid cells throughout the dermis. Higher magnification showed infiltration of blastoid neoplastic cells with scant agranular cytoplasm and prominent nucleoli. **D-I,** Immunohistological staining for CD4 **(D)**, CD56 **(E)**, CD123 **(F)**, TCL1A **(G)**, CD303 **(H)**, and CD1a **(I)**. All pictures are at 400× magnification. *H&E*, hematoxylin and eosin.

Eighteen months after the diagnosis of LCH, a painful reddish nodule developed on the scalp (Fig 2, *A*). Biopsy revealed dense infiltration of lymphoid cells throughout the dermis (Fig 2, *B*). At higher magnification, blastoid neoplastic cells with scant agranular cytoplasm and prominent nucleoli were seen (Fig 2, *C*). Immunohistochemical staining showed that the tumor cells were positive for CD4, CD56, CD123, and TCL1A, but negative for CD303 and CD1a (Figs 2, *D-I*). These findings were consistent with BPDCN. Bone marrow biopsy showed hypercellular marrow with trilineage hematopoiesis. A small number of atypical cells were observed, and these cells were positive for CD45, CD4, CD56, TCL1A, and CD123, consistent with bone marrow involvement of BPDCN. Multidrug chemotherapy was planned. However, the patient died from septic shock due to a urinary tract infection before treatment could be initiated.

**Fig 3:**
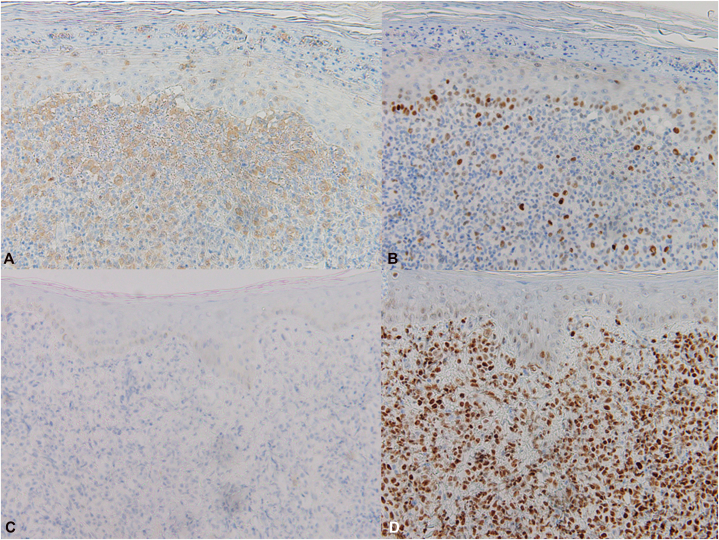
**A** and **B,** Biopsy specimens of Langerhans cell histiocytosis are stained for BRAF V600E **(A)** and c-Myc **(B)**. **C** and **D,** Biopsy specimens of blastic plasmacytoid dendritic cell neoplasm are stained for BRAF V600E **(C)** and c-Myc **(D)**. All pictures are at 400× magnification.

To evaluate molecular alterations in each tumor, immunohistochemical staining for BRAF V600E and c-Myc was performed. LCH tumor cells were positive for BRAF V600E and partially positive for c-Myc. In contrast, BPDCN tumor cells were negative for BRAF V600E and positive for c-Myc (Fig 3, *A-D*). These findings suggest that different molecular events occurred in each neoplasm.

## Discussion

Second primary malignancies are more frequent in patients with hematologic malignancies than in the general population. One cohort study reported that 32% of 132 adult patients with LCH developed additional malignancies.^6^ Furthermore, BRAF V600E mutation has been associated with an increased incidence of second primary malignancies after LCH, with a 5-year cumulative incidence of 17.3%.^7^ On the other hand, 8q24 rearrangement and Myc expression were detected in about 40% cases with BPDCN.^8^ Our patient had BRAF V600E-positive LCH and subsequently developed BPDCN.

The mechanism of secondary malignancy in LCH remains unclear. One possible explanation is the presence of a common precursor cell. Hematopoietic stem cells give rise to monocytes, which can differentiate into macrophages or dendritic cells. Dendritic cells are broadly classified into conventional dendritic cells and plasmacytoid dendritic cells. Langerhans cells belong to the conventional dendritic cell lineage, whereas BPDCN is classified as a plasmacytoid dendritic cell neoplasm in the fifth edition of the World Health Organization classification.^9^

In our case, LCH tumor cells were positive for BRAF V600E and partially positive for c-Myc, whereas BPDCN tumor cells were negative for BRAF V600E and positive for c-Myc. These results suggest that distinct molecular alterations were involved in each disease. At the same time, the sequential development of 2 dendritic cell neoplasms raises the possibility of an underlying predisposition at the level of multipotent progenitor cells.

Another possible explanation for secondary malignancy is treatment-related oncogenesis. However, our patient received only topical steroids and narrowband ultraviolet B phototherapy. In addition, BPDCN developed only 18 months after the diagnosis of LCH, making treatment-related effects less likely.

This case highlights 2 important clinical points. First, patients with LCH, especially those with BRAF V600E mutation, may have an increased risk of additional hematologic malignancies. Careful long-term follow-up is therefore important. Second, when new or rapidly growing skin nodules appear during follow-up, prompt biopsy is essential to exclude aggressive neoplasms such as BPDCN.

In conclusion, we report a rare case of BPDCN occurring after LCH. The distinct immunohistochemical findings suggest different molecular events in each tumor. This sequential occurrence may provide insight into the pathogenesis of dendritic cell neoplasms and underscores the importance of careful monitoring in patients with LCH.

## Conflicts of interest

None disclosed.
